# HOme-based Longitudinal Investigation of the multidiSciplinary Team Integrated Care (HOLISTIC): protocol of a prospective nationwide cohort study

**DOI:** 10.1186/s12877-020-01920-1

**Published:** 2020-11-27

**Authors:** Jung-Yu Liao, Ping-Jen Chen, Yu-Lin Wu, Ching-Hsia Cheng, Sang-Ju Yu, Chi-Hsien Huang, Chia-Ming Li, Ying-Wei Wang, Kai-Ping Zhang, I-Te Liu, Hiroyuki Umegaki, Jun Hamano, Masanori Mori, Irene Petersen, Elizabeth L. Sampson, Chao A. Hsiung

**Affiliations:** 1 Institute of Population Health Sciences, National Health Research Institutes, Miaoli County, 350 Taiwan; 2 Department of Family Medicine, Kaohsiung Medical University Hospital, Kaohsiung Medical University, Kaohsiung City, 807 Taiwan; 3 School of Medicine, College of Medicine, Kaohsiung Medical University, Kaohsiung City, 807 Taiwan; 4 Marie Curie Palliative Care Research Department, Division of Psychiatry, University College London, London, W1T 7NF UK; 5 Department of Nursing, St. Mary’s Junior College of Medicine, Nursing and Management, Yilan County, 266 Taiwan; 6 Department of Social Welfare, National Chung-Cheng University, Chiayi County, 621 Taiwan; 7 Home Clinic Dulan, Taitung County, 959 Taiwan; 8 Department of Community Healthcare and Geriatrics, Nagoya University Graduate School of Medicine, Nagoya, Aichi Prefecture 466-8550 Japan; 9 Department of Family Medicine, E-Da Hospital, Kaohsiung City, 824 Taiwan; 10 School of Medicine for International Students, College of Medicine, I-Shou University, Kaohsiung City, 840 Taiwan; 11 ​Department of Family Medicine, National Taiwan University Hospital, Beihu Branch, Taipei City, 108 Taiwan; 12 Health Promotion Administration, Ministry of Health and Welfare, Taipei City, 103 Taiwan; 13 Home Clinic Dulan, Taipei City, 106 Taiwan; 14 Taiwan Society of Home Health Care, Taipei City, 106 Taiwan; 15 Division of Clinical Medicine, Faculty of Medicine, University of Tsukuba, Ibaraki, 305-8575 Japan; 16 Palliative Care Team, Seirei Mikatahara General Hospital, Shizuoka, 433-8558 Japan; 17 UCL Department of Primary Care and Population Sciences, University College London, London, NW3 2PF UK; 18 Barnet Enfield and Haringey Mental Health Trust Liaison Psychiatry Team, North Middlesex University Hospital, London, UK

**Keywords:** Home health care (HHC), Cohort study, Health status, Geriatric assessment, Caregiving burden, Long-term care

## Abstract

**Background:**

The use of home health care (HHC) is increasing worldwide. This may have an impact not only on patients and their caregivers’ health but on care resource utilization and costs. We lack information on the impact of HHC on the broader dimensions of health status and care resource utilization. More understanding of the longitudinal HHC impact on HHC patients and caregivers is also needed. Moreover, we know little about the synergy between HHC and social care. Therefore, the present study aims to observe longitudinal changes in health, care resource utilization and costs and caregiving burden among HHC recipients and their caregivers in Taiwan.

**Methods:**

A prospective cohort study “Home-based Longitudinal Investigation of the Multidisciplinary Team Integrated Care (HOLISTIC)” will be conducted and 600 eligible patient-caregiver dyads will be recruited and followed with comprehensive quantitative assessments during six home investigations over two years. The measurements include physical function, psychological health, cognitive function, wellbeing, shared decision making and advance care planning, palliative care and quality of dying, caregiving burden, continuity and coordination of care, care resource utilization, and costs.

**Discussion:**

The HOLISTIC study offers the opportunity to comprehensively understand longitudinal changes in health conditions, care resource utilization and costs and caregiving burden among HHC patients and caregivers. It will provide new insights for clinical practitioners and policymakers.

**Trial registration:**

ClinicalTrials.gov Identifier is NCT04250103 which has been registered on 31st January 2020.

**Supplementary Information:**

The online version contains supplementary material available at 10.1186/s12877-020-01920-1.

## Background

Population aging means that the scope of health care delivery needs to shift from hospital-based care to home health care (HHC) particularly for older adults and other patients with chronic disease and disability [1]. HHC refers to a diverse range of health care provided by multidisciplinary healthcare professionals in patients’ homes [2]. It covers services from acute care, post-acute care and advanced treatment for chronic or terminal illnesses, providing more flexible and tailored services for patients. A growing need for HHC has led to an increased number of HHC agencies. In the United State, there were 12,200 HHC agencies and 4.5 million patients receiving HHC services in 2015–2016 [3]. In Europe, HHC is involved in the home care so that it’s not clear about the development of HHC but the growing need for home care is found [4]. Provision of HHC services is very diverse that there are numerous models and great geographic and international variation [5–7].

### HHC programs and research

HHC comprises of different programs worldwide. One of the programs is Hospital at home (HaH) which provides an option for patients to receive acute hospital care at home. Home-based primary care (HBPC) is another multidisciplinary team-based program akin to HaH, but HBPC tends to provide long-term support to high-risk, medically vulnerable patients (e.g., those suffering multiple serious chronic conditions). Both programs were found to have an impact on patients’ health and decrease costs of care [8–11]. Another program, early supported discharge (ESD), reduces the length of hospital stay and long-term dependency in patients with stroke [12].

However, studies related to the aforementioned programs focused less on the long-term changes to HHC patients’ health and disease progression. One prospective longitudinal cohort study is the Observational study of Nagoya Elderly with Home-based primary care (ONE HOME) study in Japan. Findings of the ONE HOME study suggest that HHC patients with dependent functional status had poorer family-reported quality of life (QOL), whereas nutritional status was correlated with better QOL [13]. More research is needed to understand the trajectories of comprehensive health dimensions in those receiving HHC which may influence medical utilization and costs for patients at home.

HHC patients are mainly older adults with an average age of 70 years [14] who may experience unexpected death because of multiple risk factors including frailty, comorbidity and polypharmacy. A study by Li et al. [15] found that the one-year mortality rate of HHC patients in Taiwan was 25% and significantly influenced by age and disease severity. Home deaths are increasing, especially among people with Alzheimer’s disease-related dementias, with a rate of 13.6% in 2003 to 21.9% in 2017 [16]. Therefore, programs of HHC and palliative care at home are becoming more integrated which may provide optimal care and increase the continuity of care and quality of death [17]. To promote the quality of care continuity and dying, shared decision making (SDM) and advance care planning (ACP) have highlighted the role that patients themselves have in the processes of treatment and end-of-life care and the importance of the philosophy of patient- centered care. Past HHC studies did not observe participants from illness to death or assess the inflences of SDM and ACP on quality of death. It is important to understand the causal processes underlying aging and how these potential risk factors affect healthcare costs longitudinally.

### Integration of HHC and long-term care in Taiwan

HHC has been implemented in Taiwan since 1995 and is reimbursed by the National Health Insurance program [18]. To satisfy the growing needs of HHC and provide optimal care at home, the Taiwan government integrated HHC and home palliative care into a single program “integrated home-based medical care” in 2016. HHC services include nurse and physician visits, laboratory tests, diagnosis and treatment (e.g., prescribe medication, tube or catheter replacement, and wound care) in addition to respiratory therapy and palliative care. Compared with ESD and HBPC programs, the current HHC in Taiwan provides comprehensive services for patients to address continuity of care.

In addition, the National Ten-year long-term care (LTC) Plan 2.0 has been implemented by the Taiwan government since 2017 in response to the rapidly aging population. In Taiwan LTC provides enhanced services and assists more people in need, providing not only skilled nursing, physical therapy, occupational therapy, and homemaking aid assistance, but the provision of resources and coupling of community-based services (e.g., nutritional support, living aids, transportation, and caregiver supports) to reach a larger population [19]. The financial reimbursement for LTC is from the government budget and taxes on gifts, inheritance, and tobacco.

Patients may receive medical and social care simultaneously. However, prior studies focused on either HHC or LTC and less on synergistic influences between the two on health outcomes. More studies are needed to understand if better integration of HHC and LTC may increase patients’ health status and wellbeing, and decrease the care burden.

### Aims of the current study

To address the aforementioned issues and provide insights into HHC cohort research through longitudinal methods, the HOme-based Longitudinal Investigation of the multidiSciplinary Team Integrated Care (HOLISTIC) was established and funded by National Health Research Institutes in Taiwan. The aims of the HOLISTIC study are to (1) observe the longitudinal changes of health-related outcomes, end-of-life issues, and utilization of health and social care resources of HHC recipients, (2) explore associations between HHC recipients and their caregivers and (3) investigate interaction effects between health and social care on the aforementioned evaluations.

## Methods

The HOLISTIC study is the first prospective longitudinal study of HHC in Taiwan. We will recruit eligible patients with HHC services and their caregivers and conduct assessments at baseline (T0), 3-month follow-up (T1), 6-month follow-up (T2), 12-month follow-up (T3), 18-month follow-up (T4), and 24-month follow-up (T5). This study has been approved by the Research Ethics Committee of National Health Research Institutes in Taiwan (EC1080203, EC1080203-R1) and registered on ClinicalTrials.gov (NCT04250103).

### Participants

A total of 600 eligible HHC patients and 600 caregivers will be recruited. We anticipate that HHC patients will have at least one caregiver who may be one of the patients’ relatives or an employed carer. Caregivers will be sought and enrolled where applicable. The inclusion criteria for patients and caregivers are as follows: (1) patients age 50 years and older, whereas caregivers age 20 years and older, (2) patients have consistently received home health care for 2 months, (3) Patients and caregivers can communicate with an interviewer in a familiar language, and (4) Patients with cognitive impairment are be supported by cognitively competent caregivers to communicate with an interviewer. HHC patients with a clinically predicted life expectancy of 2 months or those unwilling to give informed consent will be excluded from the study.

To ensure the study achieves the target sample, the investigators will examine the distribution of the living area and the number of participants in each type of home health care unit during recruitment. Moreover, the investigators have estimated an attrition rate of 20% over the study. Higher drop-off rates in certain sub-groups may be problematic, so investigators will monitor participant attrition.

### Sample size calculation

We will be measuring the participants at six times. Pituch and Stevens [20] suggested that when estimated effect size was small with the estimation of the average correlation of the participants’ responses = 0.5, the required sample size was 114 for each group when α was set at 0.05 and power was set at 0.8. Moreover, patients and caregivers in this study will be recruited in towns clarified to three levels of urbanization (urban, suburban and rural) and we anticipate enough participants in each sub-group. Considering a rate of 20% loss to follow-up and mortality of 28.9% among HHC patients [21], 600 patients and 600 caregivers are needed. The sample size is calculated as below:

114 × 3(three urbanization subgroups) ÷ (1–28.9%) ÷ (1–20%) = 601.

### Recruitment

Participants will be recruited by HHC teams from 18 healthcare facilities selected in two stratifications. First, towns in Taiwan were classified to three levels of urbanization (urban, suburban and rural) based on population density, ageing (population ratio of elderly people aged 65 years or older), education level (population ratio of people who graduate from college), industrialization (population ratio of agricultural workers) and distribution of medical resource (the number of physicians per 100,000 people) [22]. Second, healthcare facilities which provide HHC services will be classified into two levels (hospitals or clinics and community home care institutions).

After an invitation, eight HHC teams from three hospitals, three clinics and two community home care institutions in urban areas will participate in the study. In suburban areas, four HHC teams from a hospital, two clinics and a community home care institution will participate in the study. In rural areas, six HHC teams from five clinics and a community home care institutions will participate in the study.

At the beginning of recruitment, staff in HHC teams will briefly introduce the study protocol to patients and their caregivers who meet the criteria. If they are willing to participate in the study, our trained interviewers will contact them by phone, reconfirm their eligibility and make a home visit to explain more detailed information regarding the study. Participants will be required to sign informed consent before the initiation of the interview.

### Measures

A structured questionnaire was developed through three stages from November 2018 to August 2019. Firstly, literature reviews were made to understand the current development of HHC. We conducted a qualitative study with in-depth interviews to explore the shape and scope of HHC in Taiwan. The interviews covered healthcare providers in HHC teams and patients as well as their caregivers [23].

**Fig. 1:**
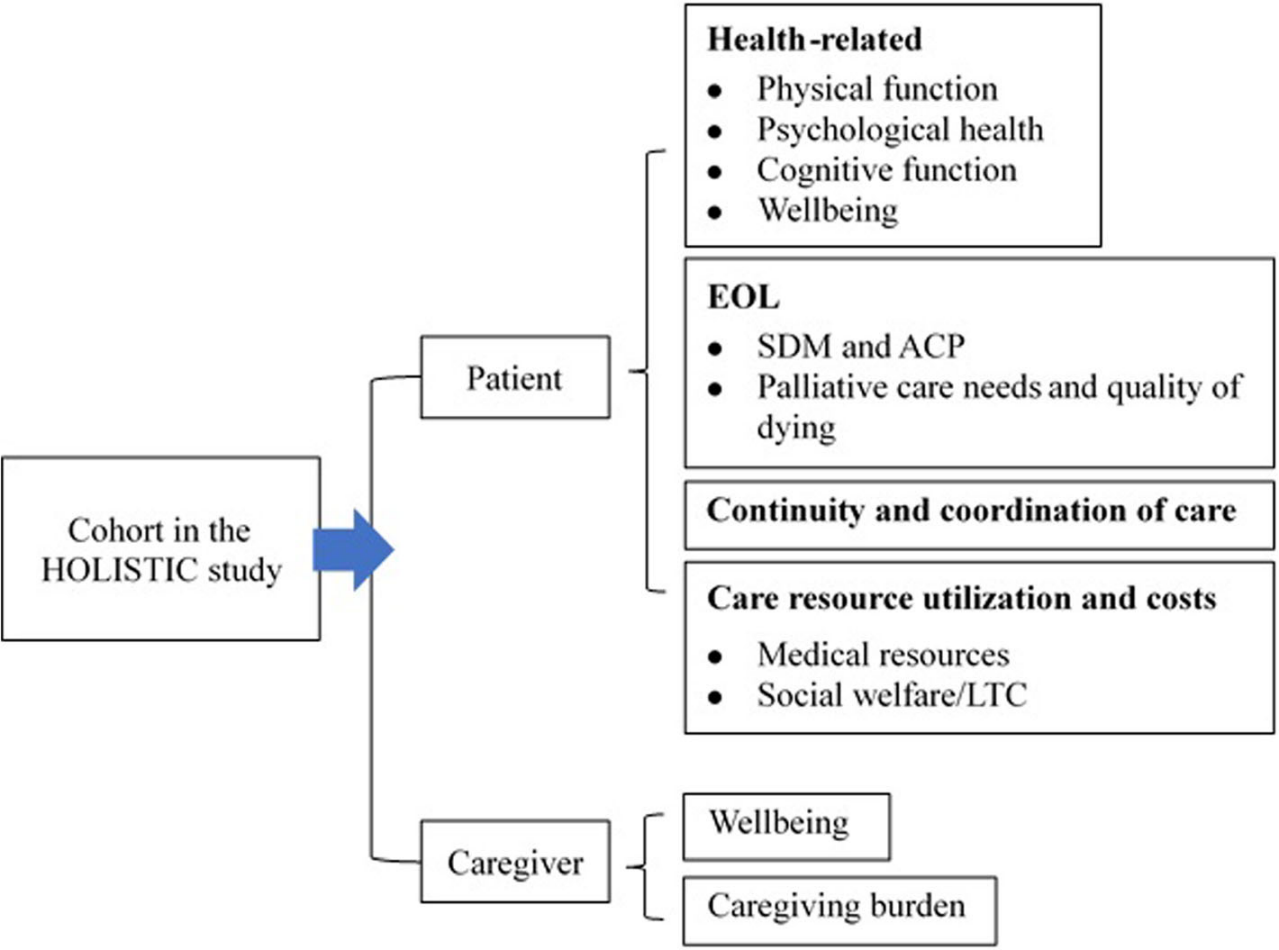
The measurement framework in the study

In the second stage, a measurement framework (Fig. 1) was developed by a workshop convened with 15 HHC providers who were divided into four groups. Based on literature reviews and perspectives from the qualitative study, four groups discussed the strengths and limitations of the current HHC program in Taiwan. This informed the measurement framework, including health outcomes, end-of-life issues, caregiving burden, continuity and coordination of care, and costs for care resource utilization (include social welfare/LTC).

The third stage was the development of a structured questionnaire based on the measurement framework. Considering the opportunity for international comparison [5], we invited international experts engaged in research on home-dwelling populations in England and Japan to participate in the development of evaluation tools. Moreover, three domestic expert panel meetings were convened to translate scales, modify questions/items with linguistic problems [24] and consider the context and culture of clinical practice in Taiwan to develop an interviewer training manual for the study. A total of 15 professionals with expertise in HHC, nutrition, palliative medicine, geriatrics and gerontology, nursing, health economics, long-term care, social welfare and public health participated in the meetings. We went through procedures for forward-backwards translations of scales/ questions which were not translated in prior studies [25]. All items in this structured questionnaire were piloted among HHC patients via face-to-face interviews for the feasibility testing. The questionnaire was finalized after modifications following discussions of the results drawn from feasibility testing.

All the measurements are shown in Table 1. Most of them will be assessed at each follow-up time point. SDM self-efficacy, ACP and needs assessment for supportive & palliative care will be assessed at every 6 months. Quality of dying will be assessed after the death of participants. Costs for medical resource utilization will be assessed at the baseline, three-month, 12-month, and 24-month follow-ups.

**Table 1:** Measurements and their instruments for patients and caregivers

Measurement	Measurement instruments	Participants	Assessment timepoint
Physical function			
Ability to perform activities of daily living	Barthel Index [26]	Patient	T0-T5
Frailty	9-point Clinical Frailty Scale [27]	Patient	T0-T5
Nutrition status	Mini Nutritional Assessment short-form [28]	Patient	T0-T5
Functionality of oral intake	Functional oral intake scale [29]	Patient	T0-T5
Risk for pressure injury	The Braden Scale for Predicting Pressure Sore Risk [30]	Patient	T0-T5
Drugs for chronic disease	Self-constructed; numbers of drug	Patient	T0-T5
Psychological health			
Depression	5-item Geriatric Depression Scale [31]	Patient	T0-T5
Neuropsychiatric status	Neuropsychiatric Inventory [32]	Patient	T0-T5
Cognitive function			
Cognitive function/memory	Brain Health Test (Chinese version, Taiwan) [33]	Patient	T0-T5
Severity of Dementia	Functional Assessment Staging Test [34]	Patient	T0-T5
Wellbeing			
QOL for patients with cognitive impairment	QOL in Alzheimer’s Disease scale [35, 36]	Patient	T0-T5
QOL for people with normal cognition	World Health Organization- Five Well-Being Index [37]	Patient, caregiver	T0-T5
	Five-level version of EuroQol five-dimensional descriptive system [38]	Patient, caregiver	T0-T5
	QOL- Home Care [39]	Patient	T0-T5
SDM and ACP			
SDM self-efficacy	Decision-making Participation Self-Efficacy Scale [40]	Patient	T0, T2-T5
ACP	4 questions from ACP engagement survey [41]	Patient	T0, T2-T5
Palliative care and quality of dying			
Symptoms	Integrated Palliative care Outcome Scale [42]	Patient	T0-T5
Needs assessment for supportive & palliative care	Supportive & Palliative Care Indicators Tool [43]	Patient	T0, T2-T5
Quality of Dying	Quality of Dying in LTC [44]	Patient	NA ^a^
Caregiving burden	Revised version of Zarit Burden interview	Caregiver	T0-T5
Continuity and coordination of care	Self-constructed; Two items for continuity from hospital to home care, and two items for coordination of home healthcare team and LTC workers, rating with a 7-point Likert-type scale	Patient	T0-T5
Care resource utilization and costs			
Medical resources	Self-constructed; Items about medical resource utilization and costs	Patient	T0-T1, T3, T5
Social welfare/LTC	Self-constructed; Items for the social resource utilization and costs	Patient	T0-T5

*QOL* quality of life, *SDM* shared decision making, *ACP* advance care planning, *LTC* long-term care

T0 = baseline; T1 = 3-month follow-up, T2 = 6-month follow-up, T3 = 12-month follow-up, T4 = 18-month follow-up, T5 = 24-month follow-up

^a^ Quality of dying will be assessed after the patient’s death

### Procedure

A home interview is estimated to take 1–1.5 h. Questionnaires (see the supplementary file) will be administered by a trained interviewer. In some cases, caregivers may support patients to complete the evaluation process (e.g., patients with impaired cognition), and interviewers will note whether the evaluation is fully completed by patients themselves or supported by caregivers.

After the baseline assessments (T0), participants will be followed up a further five times. An acceptable window for each follow-up will be 2 weeks before or after the anticipated follow-up time point. Participants will be offered 200 Taiwan dollars after they complete each home-visit interview.

Interviewers in the study will receive 6 h of training in using the interviewer training manual for the HOLISTIC study. The content of the training comprises an introduction to the study, rules of interviews, measurement instruments and background knowledge about HHC in Taiwan. In addition, principal investigators will supervise interviewers with monthly meetings to solve any problems interviewers encounter and monitor the quality of interviews and aim to decrease intra-rater biases.

### Data analysis

Double data entry will be utilized to avoid typing errors and ensure good-quality data. Before entering survey data, a research assistant will review questionnaires to manage and reduce missing data. Another two trained research assistants will be assigned to complete the first round and second round of data entry respectively. If a double-entry does not match, the researcher will review questionnaires, identify the errors and ask research assistants in charge to correct the errors. The process will be repeated until all data match in the first and second rounds.

Following that, a preliminary analysis will be used to summarise the data and describe the key features of the data for further analysis. Continuous variables will be reported as mean and standardized deviation (SD) or median and interquartile range (IQR), categorical variables as number with percentage. Comparison between the groups will be conducted using the t-test or Mann-Whitney U-test for normal and non-normal continuous data respectively, Chi-squared test or Fisher’s exact test for categorical data. The significance level will be set as 0.05.

Multiple regression analyses will be used to correct for possible confounders firstly. Generalized estimating equations will be used for continuous outcomes repeatedly measured over time to find potential time-related correlations and compare the effect between health and social care on outcomes. The interaction of group and time (group x time) will be assessed to examine whether the change over time differed between groups (e.g., gender, levels of urbanization, use of health and social care). Continuous outcomes measured only ones (e.g., quality of dying) will be analyzed using multivariable linear regression models, whereas dichotomous outcomes will be analyzed using logistic regression models.

The actor-partner interdependence model, a longitudinal model for dyadic data, will be used to explore associations between patients and caregivers. To determine the actor, partner and dyadic-level effects associated with each outcome, the dataset will be structured in a pairwise format [45]. Both the non-independence within dyads and the non-independence over time need to be accounted for. Multilevel path-analysis with fixed slopes will be used [46].

## Discussion

Little is known about the trajectories of different dimensions of health status as diseases progress toward the end-of-life and the relationships between these health dimensions in HHC research. This HOLISTIC study offers the opportunity to address this evidence gap and add to the literature. The observed health dimensions of interest include physical functions, psychological health, cognitive function, and wellbeing (e.g., QOL). Moreover, we focus on changes in shared decision making and advance care planning as well as palliative care and quality of dying to understand how these affect each other over time.

The HOLISTIC study will provide much-needed evidence about health and costs of disability and over the disease trajectory and how these differ for particular subgroups. It gives a picture on which combinations of HHC services and LTC support are most beneficial and cost-effective. For clinical practitioners, the measurements used in the study could be tools used to comprehensively understand and improve patients’ health and wellbeing. For policymakers, it will afford new insights into how HHC services influence the health of patients and caregivers in combination with LTC supports and how medical costs changes throughout the disease trajectory.

## Supplementary Information

**Additional file 1.**

## Data Availability

The datasets will be stored in a non-publically available repository. The steering committee will be grouped to manage the dataset. To ensure confidentiality, non-identifiable data will be available and dispersed to project team members on reasonable request.

## Abbreviations

ACP: Advance care planning
ESD: Early supported discharge
HHC: Home health care
HOLISTIC: HOme-based Longitudinal Investigation of the multidiSciplinary Team Integrated Care
HaH: Hospital at home
HBPC: Home-based primary care
LTC: Long-term care
ONE HOME: Observational study of Nagoya Elderly with Home-based primary care QOL Quality of life
SDM: Shared decision making
