# Supplementary material for: HOme-based Longitudinal Investigation of the multidiSciplinary Team Integrated Care (HOLISTIC): protocol of a prospective nationwide cohort study
Source: BMC Geriatr. 2020 Nov 27;20:511. doi: 10.1186/s12877-020-01920-1 (PMC7694342; doi:10.1186/s12877-020-01920-1)
Supplement: Supplementary file 1 — Additional file 1. [file 12877_2020_1920_MOESM1_ESM.doc]

Questionnaire for **HO**me-based **L**ongitudinal **I**nvestigation of the Multidi**S**ciplinary **T**eam **I**ntegrated **C**are (HOLISTIC)

[Traditional Chinese version]

**English Translation for BMC Geriatrics**

Study Registry: <https://clinicaltrials.gov/ct2/show/NCT04250103>

National Health Research Institutes, Taiwan

**Patient**

**Interviewer: (Name) Interviewee: (Code)**

**Date of interview: (yyyy/mm/dd) Starting time: (HH/MM)**

**A. Background information**

**A01. Gender:** ☐ Male ☐ Female

**A02. What year and month did you born?** (yyyy/mm)

**A03. What is the level of healthcare facility you receive home health care (HHC) services from?**

☐ Hospital ☐ Clinic ☐ Community home care institution

**A04. What is the highest grade or level of school you have completed**?

| ☐ Unable to read and write | ☐ I (incomplete) grade in elementary school |
| --- | --- |
| ☐ Elementary school | ☐ Junior high school |
| ☐ High/vocational school | ☐ Junior college |
| ☐ College/university | ☐ Graduate school/graduate institute |

**A05. What is your marital status?**

☐ Married or cohabiting ☐ Widowed ☐ Separated/ divorced ☐ Single

**A06. Who do you live with?**

| ☐ Parents | ☐ Spouse |
| --- | --- |
| ☐ Children/ their spouses | ☐ Grandchildren/ their spouses |
| ☐ Brothers and sisters/ their spouses | ☐ Relatives (uncle, untie and so on) |
| ☐ Friends | ☐ Immigrant career |
| ☐ Others: |  |

**A07. What is your religion?**

| ☐ No religion | ☐ Buddhism | ☐ Taoism |
| --- | --- | --- |
| ☐ Catholic | ☐ Christian | ☐ I-Kuan Tao |
| ☐ Others: |  |  |

**A08. Household monthly income:**

| ☐ No income | ☐ ≤ NT$20,000 | ☐ NT$20,001–40,000 |
| --- | --- | --- |
| ☐ NT$40,001–60,000 | ☐ NT$60,001–80,000 | ☐ NT$8,001–100,000 |
| ☐ NT$100,001–150,000 | ☐ NT$150,001–200,000 | ☐ ≥ NT$200,000 |

**A09. Underlying diseases (diagnosed by a physician):**

| A0901. Stroke | - No | - Yes | A0902. Dementia | - No | - Yes |
| --- | --- | --- | --- | --- | --- |
| A0903. Parkinson's disease | - No | - Yes | A0904. Spinocerebellar degeneration | - No | - Yes |
| A0905. Spinal Cord Injury | - No | - Yes | A0906. Traumatic brain injury and unconscious | - No | - Yes |
| A0907. Hip fracture | - No | - Yes | A0908. Spinal fracture | - No | - Yes |
| A0909. Hemiplegia | - No | - Yes | A0910. Depression | - No | - Yes |
| A0911. Pressure injury | - No | - Yes | A0912. Peripheral vascular disease) | - No | - Yes |
| A0913. Cancer (not spread): | - No | - Yes | A0914. Metastatic cancer: | - No | - Yes |
| A0915. Respiratory disease | - No | - Yes | A0916. Insomnia | - No | - Yes |
| A0917. Mild liver disease (hepatitis B/C, hepatic steatosis…) | - No | - Yes | A0918. Moderate/severe liver disease (liver cirrhosis, jaundice, ascites, liver transplantation, …) | - No | - Yes |
| A0919. Hypertension | - No | - Yes | A0920. Hyperlipidaemia | - No | - Yes |
| A0921. Diabetes | - No | - Yes | A0922. Diabetes complications (diabetic retinopathy, nerve damage, kidney damage, …) | - No | - Yes |
| A0923. Heart failure | - No | - Yes | A0924. Myocardial Infarction | - No | - Yes |
| A0925. Chronic kidney disease | - No | - Yes | A0926. Joint degeneration | - No | - Yes |
| 🡪 □ Stage 4 (GFR 15-29 ml/min/1.73 m^2^)  □ Stage 5 (GFR < 15 ml/min/1.73 m^2^)  □ Dialysis or kidney transplantation | | | A0927. Benign prostatic hyperplasia | - No | - Yes |
| A0928. Neurogenic bladder | - No | - Yes | A0929. Peptic ulcer disease | - No | - Yes |
| A0930. autoimmune disorder/ rheumatoid arthritis | - No | - Yes | A0931. Thyroid disease | - No | - Yes |
| A0932. Others |  | | | | |

**B. Physical function**

**B01. Ability to perform activities of daily living [Barthel Index, Traditional Chinese version]**

|  | Score | Definition |
| --- | --- | --- |
| B0101 Feeding | - 10 | Independent. The patient can feed himself a meal from a tray or table when someone puts the food within his reach. He must put on an assistive device if this is needed, cut up the food, use salt and pepper, spread butter, etc. He must accomplish this in a reasonable time. |
|  | - 5 | Some help is necessary (with cutting up food, etc., as listed above). |
|  | - 0 | Unable |
| B0102 Moving from wheelchair to bed and return (includes sitting up in bed) | - 15 | Independent in all phases of this activity. Patient can safely approach the bed in his wheelchair, lock brakes, lift footrests, move safely to bed, lie down, come to a sitting position on the side of the bed, change the position of the wheelchair, if necessary, to transfer back into it safely, and return to the wheelchair |
|  | - 10 | Either some minimal help is needed in some step of this activity or the patient needs to be reminded or supervised for safety of one or more parts of this activity. |
|  | - 5 | Patient can come to a sitting position without the help of a second person but needs to be lifted out of bed, or if he transfers with a great deal of help. |
|  | - 0 | Unable |
| B0103 Doing personal toilet | - 5 | Patient can wash hands and face, comb hair, clean teeth, and shave. He may use any kind of razor but must put in blade or plug in razor without help as well as get it from drawer or cabinet. Female patients must put on own makeup, if used, but need not braid or style hair. |
|  | - 0 | Unable |
| B0104 Getting on and off toilet | - 10 | Patient is able to get on and off toilet, fasten and unfasten clothes, prevent soiling of clothes, and use toilet paper without help. He may use a wall bar or other stable object for support if needed. If it is necessary to use a bed pan instead of a toilet, he must be able to place it on a chair, empty it, and clean it. Patient needs help because of imbalance or in handling clothes or in using toilet paper. |
|  | - 0 | Unable |
| B0105 Bathing self | - 5 | Patient may use a bath tub, a shower, or take a complete sponge bath. He must be able to do all the steps involved in whichever method is employed without another person being present. |
|  | - 0 | Unable |
| B0106 Walking on a level surface | - 15 | Patient can walk at least 50 yards without help or supervision. He may wear braces or prostheses and use crutches, canes, or a walkerette but not a rolling walker. He must be able to lock and unlock braces if used, assume the standing position and sit down, get the necessary mechanical aides into position for use, and dispose of them when he sits. (Putting on and taking off braces is scored under dressing.) |
|  | - 10 | Patient needs help or supervision in any of the above but can walk at least 50 yards with a little help. |
|  | - 5 | If a patient cannot ambulate but can propel a wheelchair independently. He must be able to go around corners, turn around, maneuver the chair to a table, bed, toilet, etc. He must be able to push a chair at least 50 yards. Do not score this item if the patient gets score for walking. |
|  | - 0 | Unable |
| B0107 Ascending and descending stairs | - 10 | Patient is able to go up and down a flight of stairs safely without help or supervision. He may and should use handrails, canes, or crutches when needed. He must be able to carry canes or crutches as he ascends or descends stairs. |
|  | - 5 | Patient needs help with or supervision of any one of the above items. |
|  | - 0 | Unable |
| B0108 Dressing and undressing | - 10 | Patient is able to put on and remove and fasten all clothing, and tie shoe laces (unless it is necessary to use adaptations for this). The activity includes putting on and removing and fastening corset or braces when these are prescribed. Such special clothing as suspenders, loafer shoes, dresses that open down the front may be used when necessary. |
|  | - 5 | Patient needs help in putting on and removing or fastening any clothing. He must do at least half the work himself. He must accomplish this in a reasonable time. Women need not be scored on use of a brassiere or girdle unless these are prescribed garments. |
|  | - 0 | Dependent |
| B0109 Continence of bowels | - 10 | Patient is able to control his bowels and have no accidents. He can use a suppository or take an enema when necessary (as for spinal cord injury patients who have had bowel training). |
|  | - 5 | Patient needs help in using a suppository or taking an enema or has occasional accidents. |
|  | - 0 | Dependent |
| B0110 Controlling bladder | - 10 | Patient is able to control his bladder day and night. Spinal cord injury patients who wear an external device and leg bag must put them on independently, clean and empty bag, and stay dry day and night. |
|  | - 5 | Patient has occasional accidents or cannot wait for the bed pan or get to the toilet in time or needs help with an external device. |
|  | - 0 | Dependent |

Source: Mahoney FI, Barthel DW. Functional evaluation: the Barthel Index: a simple index of independence useful in scoring improvement in the rehabilitation of the chronically ill. Md State Med J 1965;14:61–5.

**B02. Frailty [****Clinical Frailty Scale, Traditional Chinese version]**

**Which of the following also applies to you?**


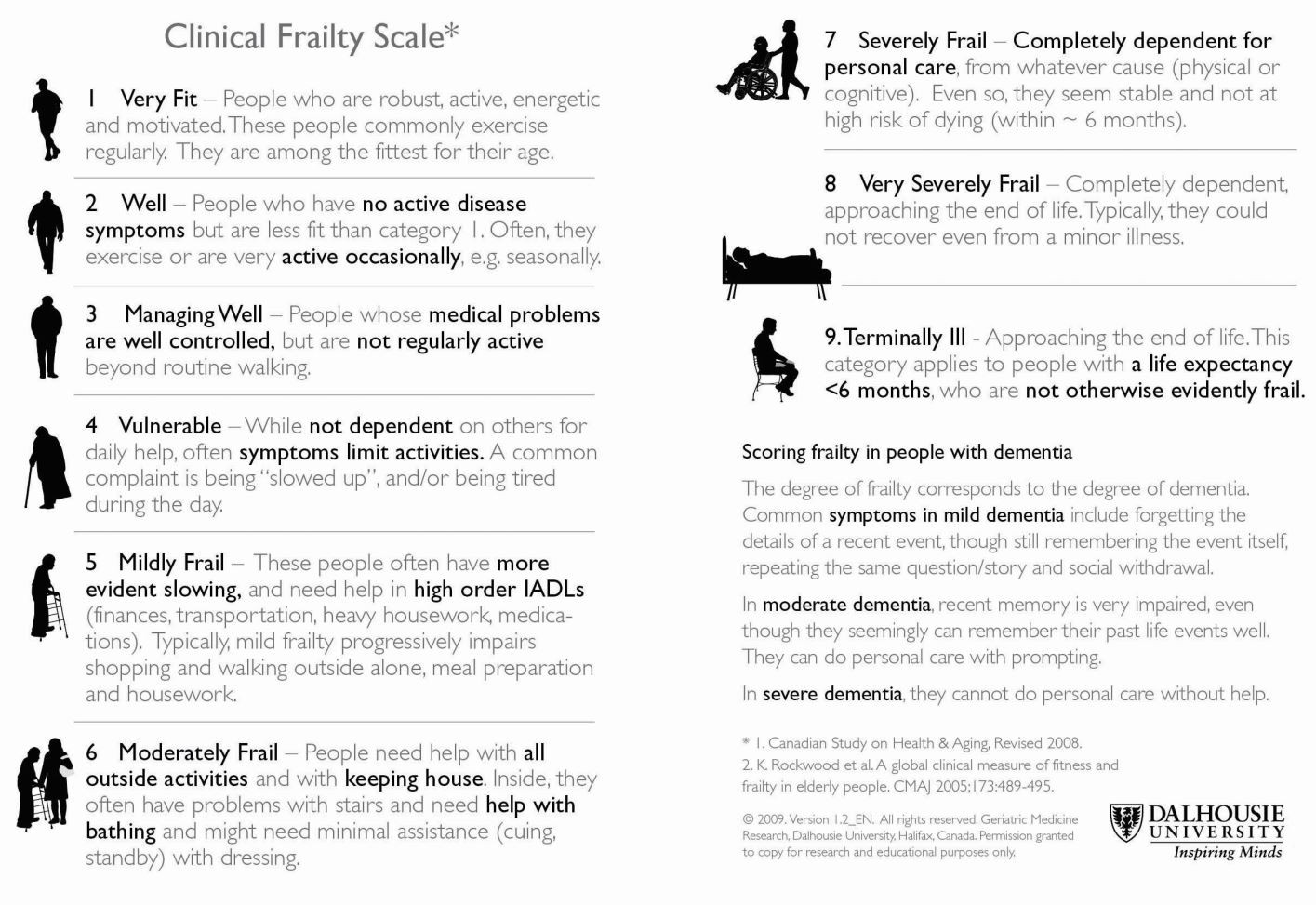


Source: Rockwood K, Song X, MacKnight C, Bergman H, Hogan DB, McDowell I et al. A global clinical measure of fitness and frailty in elderly people. Can Med Assoc J 2005;173(5):489-95.

**B03. Nutrition status [****Mini Nutritional Assessment short-form, Traditional Chinese version]**

| B0301 | Has food intake declined over the past three months due to loss of appetite, digestive problems, chewing or swallowing difficulties? | - Severe decrease in food intake - Moderate decrease in food intake - No decrease in food intake |
| --- | --- | --- |
| B0302 | Involuntary weight loss during the last 3 months? | - Weight loss greater than 3 kg - Does not know - Weight loss between 1 and 3 kg - No weight loss |
| B0303 | Mobility?  *EX. Ask patient “How would you describe your current mobility?”* | - Bed or chair bound - Able to get out of bed/chair, but does not go out - Goes out |
| B0304 | Has the patient suffered psychological stress or acute disease in the past three months? | - Yes - No |
| B0305 | Neuropsychological problems?  *Note:* The patient’s caregiver, nursing staff or medical record can provide information about the severity of the patient’s neuropsychological problems (dementia). | - Severe dementia or depression - Mild dementia - No psychological problems |
| B0306 | Body mass index (BMI)? | Height: Weight: |

Source: Kaiser MJ, Bauer JM, Ramsch C, Uter W, Guigoz Y, Cederholm T et al. Validation of the Mini Nutritional Assessment Short-Form (MNA®-SF): A practical tool for identification of nutritional status. J Nutr Health Aging 2009;13(9):782.

**B04. Functionality of oral intake [** **Functional oral intake scale, Traditional Chinese version]**

|  | Level 1: Nothing by mouth. |
| --- | --- |
|  | Level 2: Tube dependent with minimal attempts of food or liquid. |
|  | Level 3: Tube dependent with consistent oral intake of food or liquid. |
|  | Level 4: Total oral diet of a single consistency. |
|  | Level 5: Total oral diet with multiple consistencies, but requiring special preparation or compensations. |
|  | Level 6: Total oral diet with multiple consistencies without special preparation, but with specific food limitations. |
|  | Level 7: Total oral diet with no restrictions. |

Source: Crary MA, Mann GDC, Groher ME. Initial psychometric assessment of a functional oral intake scale for dysphagia in stroke patients. Arch Phys Med Rehabil 2005;86(8):1516-20.

**B05. Risk for pressure injury [** **Braden Scale, Traditional Chinese version]**

**Which of the following applies to you? Please write the applicable option to each box below.**

**
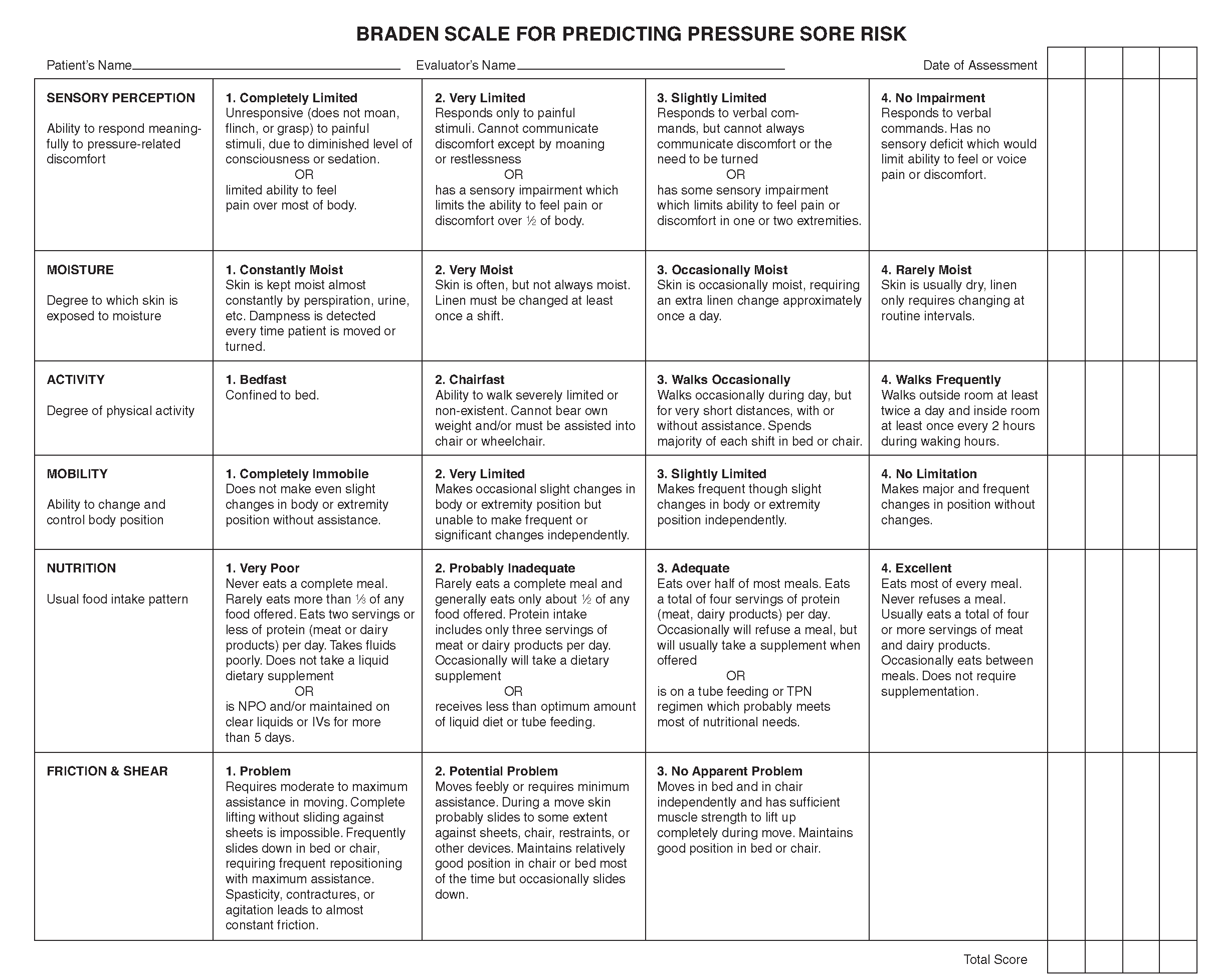
**

Resource: Bergstrom N. The Braden Scale for predicting pressure sore risk. Nurs Res 1987;36(4):205-10.

**B06. Drugs for chronic disease**

B0601. Who is in charge of prescribing the current medicine for you?

☐ HHC physician ☐ Physicians in clinics/hospitals ☐ Both

B0602. How many regular medications do you take (≥ 28 days)?

*Note: please copy and paste the prescriptions with the permission of patient*

**C. Psychological health**

**C01. Depression [5-item** **Geriatric Depression Scale, Traditional Chinese version]**

| C0101. Are you basically satisfied with your life? | - No | - Yes |
| --- | --- | --- |
| C0102. Do you often get bored? | - No | - Yes |
| C0103. Do you often feel helpless? | - No | - Yes |
| C0104. Do you prefer to stay at home rather than going out and doing new things? | - No | - Yes |
| C0105. Do you feel pretty worthless the way you are now? | - No | - Yes |

Source: Hoyl MT, Alessi CA, Harker JO, Josephson KR, Pietruszka FM, Koelfgen M et al. Development and testing of a five‐item version of the Geriatric Depression Scale. J Am Geriatr Soc 1999;47(7):873-8.

**C02. Neuropsychiatric status [** **Neuropsychiatric Inventory, Traditional Chinese version]**

*Frequency: 1=seldom, 2=sometimes, 3=usually, 4=always*

*Severity; 1=mild, 2=moderate, 3=severe*

|  |  |  | **Frequency** | **Severity** |
| --- | --- | --- | --- | --- |
| **Delusions** | - No | - Yes | **1 2 3 4** | **1 2 3** |
| **Hallucinations** | - No | - Yes | **1 2 3 4** | **1 2 3** |
| **Agitation/ Aggression** | - No | - Yes | **1 2 3 4** | **1 2 3** |
| **Depression/ Dysphoria** | - No | - Yes | **1 2 3 4** | **1 2 3** |
| **Anxiety** | - No | - Yes | **1 2 3 4** | **1 2 3** |
| **Elation/ Euphoria** | - No | - Yes | **1 2 3 4** | **1 2 3** |
| **Apathy/ indifference** | - No | - Yes | **1 2 3 4** | **1 2 3** |
| **Disinhibition** | - No | - Yes | **1 2 3 4** | **1 2 3** |
| **Irritability/ Lability** | - No | - Yes | **1 2 3 4** | **1 2 3** |
| **Moto disturbance** | - No | - Yes | **1 2 3 4** | **1 2 3** |

Source: Cummings JL, Mega M, Gray K, Rosenberg-Thompson S, Carusi DA, Gornbein J. The Neuropsychiatric Inventory: comprehensive assessment of psychopathology in dementia. Neurology 1994;44(12):2308.

**D. Cognitive function**

**D01. Cognitive function/memory [Brain Health Test, Traditional Chinese version]**


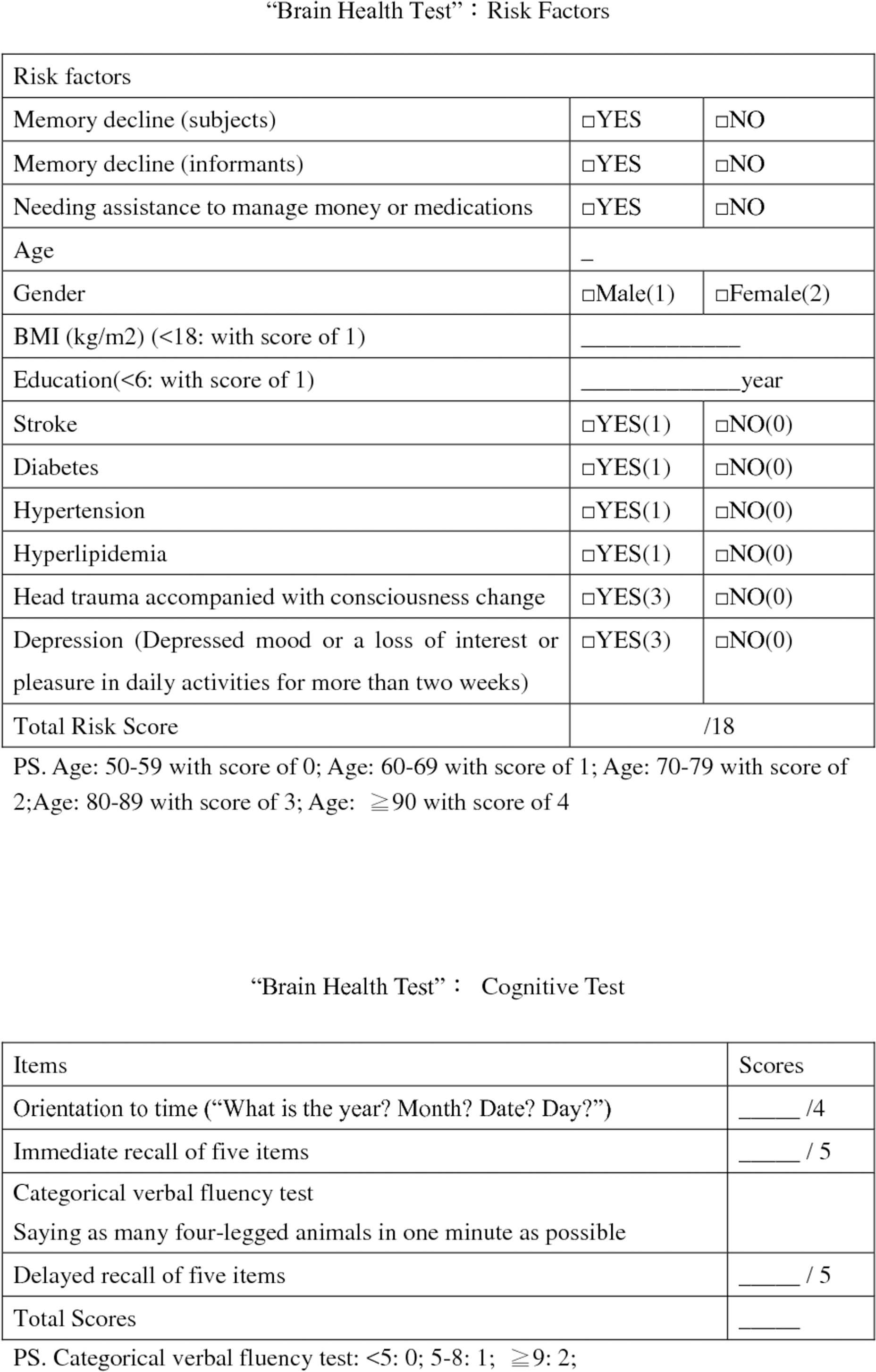


Source: Tsai PH, Liu JL, Lin KN, Chang CC, Pai MC, Wang WF et al. Development and validation of a dementia screening tool for primary care in Taiwan: Brain Health Test. PLoS One 2018;13(4).

**D02. Severity of Dementia [****Functional Assessment Staging Test, Traditional Chinese version]**

**Which of the following applies to you?**

1. No difficulty either subjectively or objectively.

2. Complains of forgetting location of objects. Subject work difficulties.

3. Decreased job functioning evident to co-workers. Difficulty in traveling to new locations. Decreased organizational capacity.*

4. Decreased ability to perform complex tasks, e.g., planning dinner for guests, handling personal finances (such as forgetting to pay bills), difficulty marketing, etc.

5. Requires assistance in choosing proper clothing to wear for the day, season or occasion, e.g., Patient may wear the same clothing repeatedly, unless supervised.*

6. a. Improperly putting on clothing without assistance or cueing (e.g., may put street clothes on overnight clothes, or put shoes on wrong feet, or have difficulty buttoning clothing) occasionally or more frequently over the past weeks.

6b. Unable to bathe properly (e.g., difficulty adjusting bath-water temperature) occasionally or more frequently over the last weeks.

6c. Inability to handle mechanics of toileting (e.g., forgets to flush the toilet, does not wipe properly or properly dispose of toilet tissue) occasionally or more frequently over the past weeks.

6d. Urinary incontinence (occasionally or more frequently over the past weeks)*.

6e. Fecal incontinence (occasionally or more frequently over the past weeks)*.

7. a. Ability to speak limited to approximately a half a dozen intelligible different words or fewer, in the course of an average day or in the course of an intensive interview.

7b. Speech ability is limited to the use of a single intelligible word in an average day or in the course of an intense interview (the person may repeat the word over and over).

7c. Ambulatory ability is lost (cannot walk without personal assistance).

7d. Cannot sit up without assistance (e.g., the individual will fall over if there are not lateral rests (arms) on the chair).

7e. Loss of ability to smile.

7f. Loss of ability to hold head up independently.

Source: Sclan SG, Reisberg B. Functional assessment staging (FAST) in Alzheimer's disease: reliability, validity, and ordinality. Int Psychogeriatr 1992;4(3):55-69.

**E.** **Wellbeing**

**E01. Quality of life (QOL) for patients with cognitive impairment**

E0101. QOL in Alzheimer's Disease scale [Traditional Chinese version]

*I want to ask you some questions about your quality of life and have you rate different aspects of your life using one of four words: poor, fair, good, or excellent. Point to each word (poor, fair, good, and excellent) on the form as you say it.*


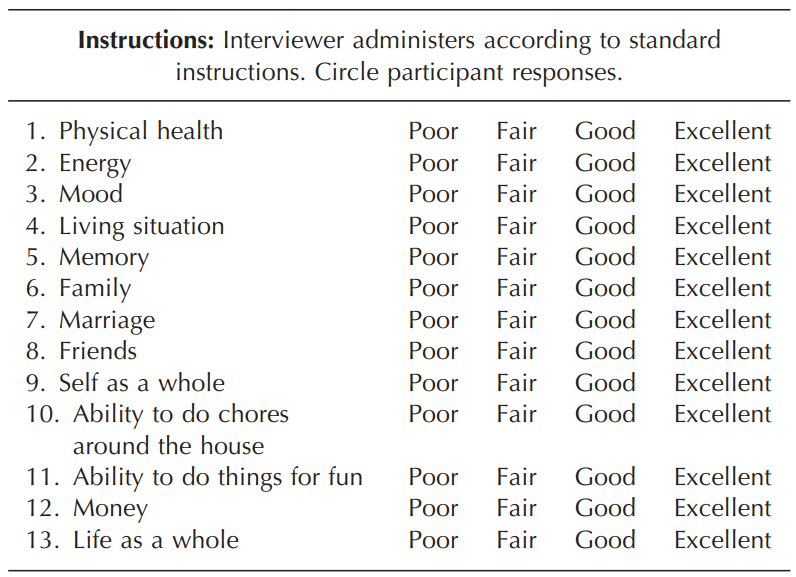


Source: Logsdon RG, Gibbons LE, McCurry SM, Teri L. Assessing quality of life in older adults with cognitive impairment. Psychosom Med 2002;64(3):510-9.

E0102. World Health Organization- Five Well-Being Index [Traditional Chinese version]

*Example: If you have felt cheerful and in good spirits more than half of the time during the last two weeks, put a tick in the box with the number 3 in the upper right corner.*


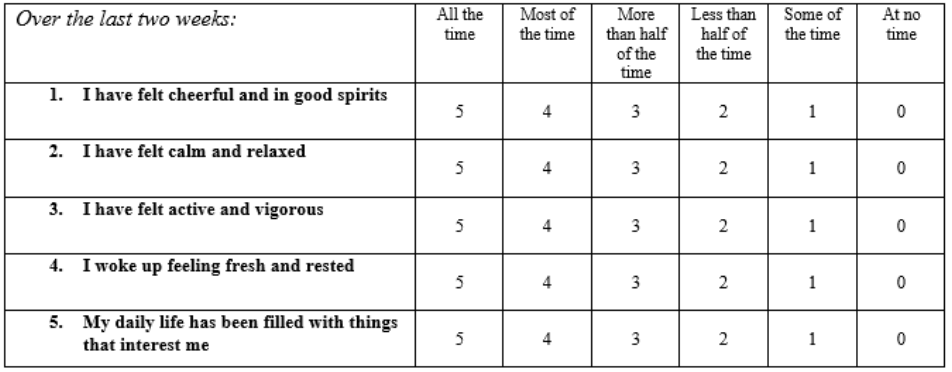


Source: Heun R, Bonsignore M, Barkow K, Jessen F. Validity of the five-item WHO Well-Being Index (WHO-5) in an elderly population. Eur Arch Psychiatry Clin Neurosci 2001;251(2):27-31.

**E02. QOL for people with normal cognition**

**E0201. Five-level version of EuroQol five-dimensional descriptive system [Traditional Chinese version]**

| Under each heading, please tick the ONE box that best describes your health TODAY | |
| --- | --- |
| E0201A.  MOBILITY | 1□ I have no problems in walking about  2□ I have slight problems in walking about  3□ I have moderate problems in walking about  4□ I have severe problems in walking about  5□ I am unable to walk about |
| E0201B.  SELF-CARE | 1□ I have no problems with washing or dressing myself  2□ I have slight problems with washing or dressing myself  3□ I have moderate problems with washing or dressing myself  4□ I have severe problems with washing or dressing myself  5□ I am unable to wash or dress myself |
| E0201C.  USUAL ACTIVITIES  (e.g. work, study, housework, family or leisure activities | 1□ I have no problems doing my usual activities  2□ I have slight problems doing my usual activities  3□ I have moderate problems doing my usual activities  4□ I have severe problems doing my usual activities  5□ I am unable to do my usual activities |
| E0201D.  PAIN/DISCOMFORT | 1□ I have no pain or discomfort  2□ I have slight pain or discomfort  3□ I have moderate pain or discomfort  4□ I have severe pain or discomfort  5□ I have extreme pain or discomfort |
| E0201E.  ANXIETY/DEPRESSION | 1□ I am not anxious or depressed  2□ I am slightly anxious or depressed  3□ I am moderately anxious or depressed  4□ I am severely anxious or depressed  5□ I am extremely anxious or depressed |

We would like to know how good or bad your health is TODAY.

- This scale is numbered from 0 to 100.
- 100 means the best health you can imagine. 0 means the worst health you can imagine.
- Mark an X on the scale to indicate how your health is TODAY.

|  |
| --- |

- Now, please write the number you marked on the scale in the box below.

**
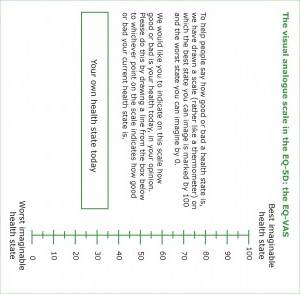
**

0 10 20 30 40 50 60 70 80 90 100

The worst health you can imagine The best health you can imagine

Source: Herdman M, Gudex C, Lloyd A, Janssen M, Kind P, Parkin D et al. Development and preliminary testing of the new five-level version of EQ-5D (EQ-5D-5L). Qual Life Res 2011;20(10):1727-36.

**E0202. World Health Organization- Five Well-Being Index [Traditional Chinese version]**

*Example: If you have felt cheerful and in good spirits more than half of the time during the last two weeks, put a tick in the box with the number 3 in the upper right corner.*


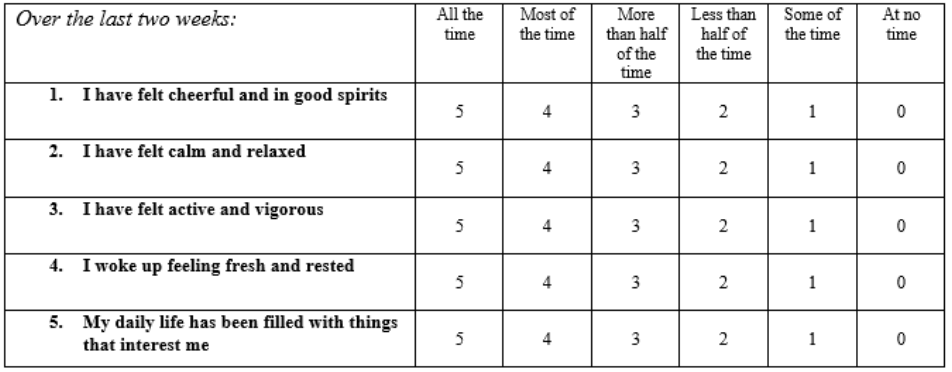


Source: Heun R, Bonsignore M, Barkow K, Jessen F. Validity of the five-item WHO Well-Being Index (WHO-5) in an elderly population. Eur Arch Psychiatry Clin Neurosci 2001;251(2):27-31.

**E0203. QOL- Home Care (from ONE-HOME study) [Traditional Chinese version]**

|  |  | Yes | No | Do not know |
| --- | --- | --- | --- | --- |
| E0203A | Do you have peace of mind? | 0□ | 2□ | 1□ |
| E0203B | Do you feel satisfied with your life when you reﬂect on it? | 0□ | 2□ | 1□ |
| E0203C | Do you have someone that you spend time talking with? | 0□ | 2□ | 1□ |
| E0203D | Are you satisﬁed with the home care service system? | 0□ | 2□ | 1□ |

Source: Kamitani H, Umegaki H, Okamoto K, Kanda S, Asai A, Maeda K et al. Development and validation of a new quality of life scale for patients receiving home‐based medical care: The Observational Study of Nagoya Elderly with Home Medical Care. Geriatr Gerontol Int 2017;17(3):440-8.

**F.** **Shared decision making and advance care planning**

**F01. Decision-making Participation Self-efficacy scale [Traditional Chinese version]**

*If at this time, you and your follow-up care doctor had to make any medical decisions about your follow-up treatment/care plan (e.g., cancer care)*, how confident are you that you would be able to…

|  | Not at all confident | A little confident | Somewhat confident | Very confident | Completely confident |
| --- | --- | --- | --- | --- | --- |
| F0101. Take part in a detailed discussion with your doctor about the different available options | 1 | 2 | 3 | 4 | 5 |
| F0102. Let your doctor know if you had any concerns or questions about his or her recommendation | 1 | 2 | 3 | 4 | 5 |
| F0103. Tell your doctor about the option you would prefer | 1 | 2 | 3 | 4 | 5 |
| F0104. Work out any differences of opinion with your doctor, should they exist | 1 | 2 | 3 | 4 | 5 |
| F0105. Take responsibility for making the final decision | 1 | 2 | 3 | 4 | 5 |

Source: Arora NK, Weaver KE, Clayman ML, Oakley-Girvan I, Potosky AL. Physicians’ decision-making style and psychosocial outcomes among cancer survivors. Patient Educ Couns 2009;77(3):404-12.

**F02. Advance Care Planning Engagement Survey (four-item) [Traditional Chinese version]**

*Introduction* We will ask about your experiences and opinions. We may ask about things that you have already done, or have not thought about at all. Just answer as honestly as you can.

| **1. Medical Decision Makers**  The following question asks about medical decision makers. A medical decision maker is a family member or friend who can make decisions for you if you were to become too sick to make your own decisions. Remember, please give us your honest opinions and there are no right or wrong answers. | |
| --- | --- |
| 1. How ready are you to SIGN OFFICIAL PAPERS naming a person or group of people to make medical decisions for you? | |
| 1 □ I have never thought about it  2 □ I have thought about it, but I am not ready to do it  3 □ I am thinking about doing it in the next 6 months  4 □ I am definitely planning to do it in the next 30 days  5 □ I have already done it | 8 □ Not sure  9 □ Refused |
| OPTIONAL:  If answered, “I have already done it,” then ask “When did you do this?  1 □ <6 months  2 □ ≥6months  99 □ NA | 8 □ Not sure  9 □ Refused |
| **2. What Matters Most in Life**  The following questions are about specific medical treatments that people may or may never want if they were very sick or at the end of their life. For instance, some people know they would want to be on a breathing machine. Other people know they would never want to be on a breathing machine. Please give us your honest opinions to the following questions about medical treatments. There are no right or wrong answers. | |
| 1. How ready are you to talk to your DECISION MAKER about the kind of medical care you would want if you were very sick or near the end of life? | |
| 1 □ I have never thought about it  2 □ I have thought about it, but I am not ready to do it  3 □ I am thinking about doing it in the next 6 months  4 □ I am definitely planning to do it in the next 30 days  5 □ I have already done it | 8 □ Not sure  9 □ Refused |
| OPTIONAL:  If answered, “I have already done it,” then ask “When did you do this?  1 □ <6 months  2 □ ≥6months  99 □ NA | 8 □ Not sure  9 □ Refused |
| 1. How ready are you to talk to your DOCTOR about the kind of medical care you would want if you were very sick or near the end of life? | |
| 1 □ I have never thought about it  2 □ I have thought about it, but I am not ready to do it  3 □ I am thinking about doing it in the next few visits  4 □ I am definitely planning to do it at the next visit  5 □ I have already done it | 8 □ Not sure  9 □ Refused |
| OPTIONAL:  If answered, “I have already done it,” then ask “When did you do this?  1 □ <6 months  2 □ ≥6months  99 □ NA | 8 □ Not sure  9 □ Refused |
| 1. How ready are you to SIGN OFFICIAL PAPERS putting your wishes about the kind of medical care you would want if you were very sick or near the end of life? | |
| 1 □ I have never thought about it  2 □ I have thought about it, but I am not ready to do it  3 □ I am thinking about doing it in the next 6 months  4 □ I am definitely planning to do it in the next 30 days  5 □ I have already done it | 8 □ Not sure  9 □ Refused |
| OPTIONAL:  If answered, “I have already done it,” then ask “When did you do this?  1 □ <6 months  2 □ ≥6months  99 □ NA | 8 □ Not sure  9 □ Refused |

**G.** **Palliative care and quality of dying**

**G01. Symptoms [Integrated Palliative care Outcome Scale, Traditional Chinese version]**

Over the past seven days, have you ever experienced any symptoms making you feel uncomfortable?

|  |
| --- |

Source: Schildmann EK, Groeneveld EI, Denzel J, Brown A, Bernhardt F, Bailey K et al. Discovering the hidden benefits of cognitive interviewing in two languages: The first phase of a validation study of the Integrated Palliative care Outcome Scale. Palliat Med 2016;30(6):599-610.

**G02. Needs assessment for supportive & palliative care [Supportive & Palliative Care Indicators Tool, Traditional Chinese version]**


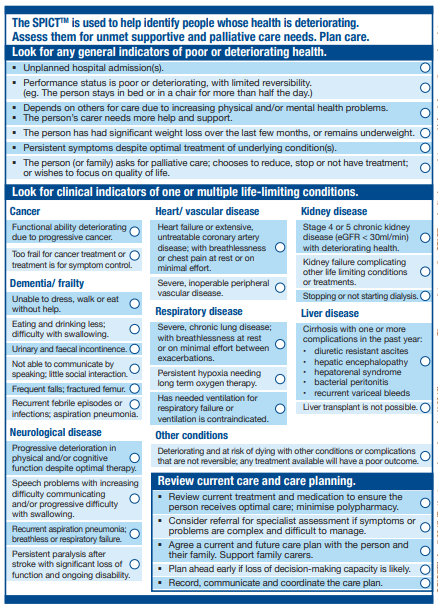


Source: Highet G, Crawford D, Murray SA, Boyd K. Development and evaluation of the Supportive and Palliative Care Indicators Tool (SPICT): a mixed-methods study. BMJ Support Palliat Care 2014;4(3):285-90. SPICT website <https://www.spict.org.uk/the-spict/>

**G03. Quality of Dying [ Quality of dying in long‐term care, Traditional Chinese version]** *assessed after a patient’s death

*1= None, 2=A little, 3=Moderate, 4=Almost, 5=Totally*

| G03A | Patient was kept clean. | 1 2 3 4 5 |
| --- | --- | --- |
| G03B | Patient received compassionate physical touch daily. | 1 2 3 4 5 |
| G03C | Patient’s dignity was maintained. | 1 2 3 4 5 |
| G03D | Patient’s physician knew him/her as a whole person. | 1 2 3 4 5 |
| G03E | There was a nurse or aide with whom the patient felt comfortable. | 1 2 3 4 5 |
| G03F | Patient was able to retain his/her sense of humor. | 1 2 3 4 5 |
| G03G | Patient indicated he/she was prepared to die. | 1 2 3 4 5 |
| G03H | Patient appeared to be at peace. | 1 2 3 4 5 |
| G03I | Patient had treatment preferences in writing. | 1 2 3 4 5 |
| G03J | Patient had named a decision-maker in the event that he/she was no longer able to make decisions. | 1 2 3 4 5 |
| G03K | Patient had funeral arrangements planned. | 1 2 3 4 5 |

Source: Munn JC, Zimmerman S, Hanson LC, Williams CS, Sloane PD, Clipp EC et al. Measuring the quality of dying in long‐term care. J Am Geriatr Soc 2007;55(9):1371-9.

| G03L | Date of death: (yyyy/mm/dd) |
| --- | --- |
| G03M | Place of death:  (1: PCU/Hospice, 2: General ward, 3: Own home, 4: Care/nursing home, 5: Home of a relative/friend, 6: Other (please note) 7: Unknown) |

**H.** **Continuity and coordination of care**

*6=Strongly agree, 5=Agree, 4=Somewhat agree, 3=Somewhat disagree, 2=Disagree, 1=Strongly disagree, 0=Unknown*

| **H01. Continuity of care provided by staffs in home healthcare teams and staffs in the hospitals** | | |
| --- | --- | --- |
| H01A | Did you perceive the physicians in home healthcare teams and the physicians in hospitals were familiar with each other? | 6 5 4 3 2 1 0 |
| H01B | Did you perceive the other healthcare professionals such as nurses or social workers in home healthcare teams and the counterpart in hospitals were familiar with each other? | 6 5 4 3 2 1 0 |
| **H02. Coordination of care among staffs in home healthcare teams** | | |
| H02A | Did you perceive the coordination of care between the physicians and other healthcare professionals such as nurses or social workers in home healthcare teams? | 6 5 4 3 2 1 0 |
| H02B | Did you perceive the coordination of care between the staffs in long-term care teams such as care managers or care aides, and staffs in home healthcare teams? | 6 5 4 3 2 1 0 |

**I. Care resource utilization and costs**

**I01. When did you last go to the doctor/go to the hospital?** (yyyy/mm/dd)

**I02. Where and why did you last go to the doctor?**

**I03. How long and how much did it take in the last time you went to the doctor/ hospital?**

**I04. Did anyone accompany you to the doctor/ hospital last time?**

**□ No □Yes**

Please describe their occupation and estimate the time and wage they took/ loss

**I05 Please estimate your medical resource utilization and cost before and after receiving home health care (HHC) services.**

|  | The last six months before receiving HHC services. | From receiving HHC services till now |
| --- | --- | --- |
| Average monthly number of going to the doctor/ hospital |  |  |
| Average monthly co-payment after the cover of National Health Insurance |  |  |
| Average monthly out-of-pocket expense and cost of care-related goods/ items (Please mention the cost for each one) |  |  |
| Average monthly cost for manpower |  |  |
| Average monthly cost of transportation for the medical care |  |  |

**I06.** Do you have any identification below?

| ☐ None | ☐ Low-income households | ☐ Mid-income households |
| --- | --- | --- |
| ☐ Disability | ☐ Veteran | ☐ Indigenous |

**I07. What long-term care services do you use?**

| - Physical care services | - No | - Yes |
| --- | --- | --- |
| - Daily life care services | - No | - Yes |
| - Domestic chore services | - No | - Yes |
| - Food and nutritional services | - No | - Yes |
| - Auxiliary appliance services | - No | - Yes |
| - Necessary home facility adjustment and improvement services | - No | - Yes |
| - Emergency rescue services | - No | - Yes |
| - Services for prevention of other or aggravated disability conditions | - No | - Yes |
| - Temporary housing services | - No | - Yes |
| - Transportation services | - No | - Yes |
| - Social participation services | - No | - Yes |
| - Home Nursing | - No | - Yes |
| - Home/Community Rehabilitation | - No | - Yes |
| - Individual Service Plan | - No | - Yes |
| - Services for prevention of other or aggravated disability conditions | - No | - Yes |
| - Others: | | |

**End time: (HH/MM)**

**Caregiver**

**Interviewer: (name) Interviewee: (code)**

**Date of interview: (yyyy/mm/dd) Starting time: (HH/MM)**

**A. Background information**

**A01. Gender:** ☐ Male ☐ Female

**A02. What year and month did you born?**  (yyyy/mm)

**A03. Where do you live?**

☐ Live with the patient ☐ Live nearby the patient’s home ☐ live another place

**A06. What relationship between you and the patient you care for**?

| ☐ Spouse | ☐ Son | ☐ Daughter-in-law |
| --- | --- | --- |
| ☐ Daughter | ☐ Grandson | ☐ Granddaughter |
| ☐ Father | ☐ Mother | ☐ Brothers |
| ☐ Sisters | ☐ father-in-law/ mother-in-low | ☐ Long-term care worker |
| ☐ Immigrant career | ☐ Employed specialized nurse/career | ☐ Relatives |
| ☐ Friend | ☐ Others: |  |

**B. Quality of Life**

**B01. Five-level version of EuroQol five-dimensional descriptive system [Traditional Chinese version]**

| Under each heading, please tick the ONE box that best describes your health TODAY | |
| --- | --- |
| E01A.  MOBILITY | 1□ I have no problems in walking about  2□ I have slight problems in walking about  3□ I have moderate problems in walking about  4□ I have severe problems in walking about  5□ I am unable to walk about |
| E01B.  SELF-CARE | 1□ I have no problems with washing or dressing myself  2□ I have slight problems with washing or dressing myself  3□ I have moderate problems with washing or dressing myself  4□ I have severe problems with washing or dressing myself  5□ I am unable to wash or dress myself |
| E01C.  USUAL ACTIVITIES  (e.g. work, study, housework, family or leisure activities | 1□ I have no problems doing my usual activities  2□ I have slight problems doing my usual activities  3□ I have moderate problems doing my usual activities  4□ I have severe problems doing my usual activities  5□ I am unable to do my usual activities |
| E01D.  PAIN/DISCOMFORT | 1□ I have no pain or discomfort  2□ I have slight pain or discomfort  3□ I have moderate pain or discomfort  4□ I have severe pain or discomfort  5□ I have extreme pain or discomfort |
| E01E.  ANXIETY/DEPRESSION | 1□ I am not anxious or depressed  2□ I am slightly anxious or depressed  3□ I am moderately anxious or depressed  4□ I am severely anxious or depressed  5□ I am extremely anxious or depressed |

**Source:**

We would like to know how good or bad your health is TODAY.

- This scale is numbered from 0 to 100.
- 100 means the best health you can imagine. 0 means the worst health you can imagine.
- Mark an X on the scale to indicate how your health is TODAY.

|  |
| --- |

- Now, please write the number you marked on the scale in the box below.

**
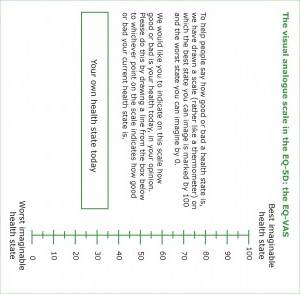
**

0 10 20 30 40 50 60 70 80 90 100

The worst health you can imagine The best health you can imagine

Source: Herdman M, Gudex C, Lloyd A, Janssen M, Kind P, Parkin D et al. Development and preliminary testing of the new five-level version of EQ-5D (EQ-5D-5L). Qual Life Res 2011;20(10):1727-36.

**B02. World Health Organization- Five Well-Being Index [Traditional Chinese version]**

*Example: If you have felt cheerful and in good spirits more than half of the time during the last two weeks, put a tick in the box with the number 3 in the upper right corner.*


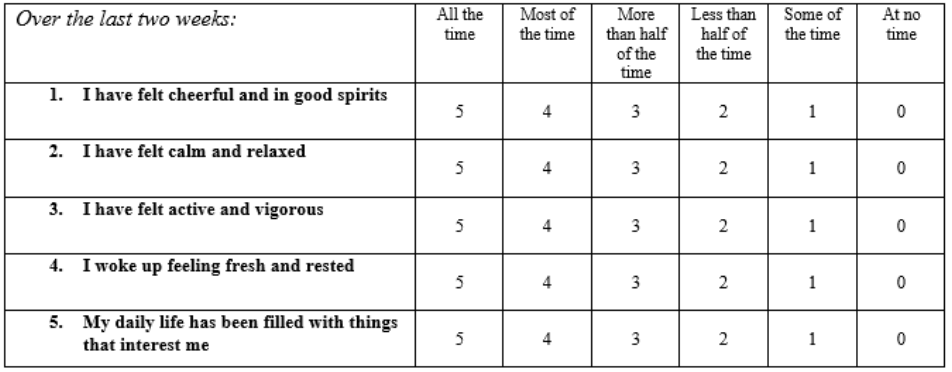


Source: Heun R, Bonsignore M, Barkow K, Jessen F. Validity of the five-item WHO Well-Being Index (WHO-5) in an elderly population. Eur Arch Psychiatry Clin Neurosci 2001;251(2):27-31.

**C.** **Caregiving burden [Revised version of Zarit Burden Interview, Traditional Chinese version]**

***0=Neve, 1=Rarely, 2=Sometimes, 3=Usually, 4=Always***

|  | *Do you feel…* |  |
| --- | --- | --- |
| C01A | You don’t have enough time for yourself? | 0 1 2 3 4 |
| C01B | Stressed between caring and meeting other responsibilities? | 0 1 2 3 4 |
| C01C | Angry when around your relative? | 0 1 2 3 4 |
| C01D | Your relative affects your relationship with others in a negative way? | 0 1 2 3 4 |
| C01E | Strained when are around your relative? | 0 1 2 3 4 |
| C01F | Your health has suffered because of your involvement with your relative? | 0 1 2 3 4 |
| C01G | You don’t have as much privacy as you would like, because of your relative? | 0 1 2 3 4 |
| C01H | Your social life has suffered because you are caring for your relative? | 0 1 2 3 4 |
| C01I | You have lost control of your life since your relative’s illness? | 0 1 2 3 4 |
| C01J | Uncertain about what to do about relative? | 0 1 2 3 4 |
| C01K | You should be doing more for your relative? | 0 1 2 3 4 |
| C01L | You could do a better job in caring for your relative? | 0 1 2 3 4 |

**End time: (HH/MM)**
